# Evaluation of the Impact of Digital Dentistry on the Precision of Implant Placement and Prosthesis Fabrication: An In-Vitro Study

**DOI:** 10.7759/cureus.60389

**Published:** 2024-05-15

**Authors:** Subhash Sonkesriya, Reshma Kulkarni, Sukanta K Satapathy, Shabna Fathima, Vishnu Thomas, Praveen Gangadharappa

**Affiliations:** 1 Department of Prosthodontics, Government College of Dentistry, Indore, Indore, IND; 2 Department of Prosthodontics, Government Dental College and Research Institute Bangalore, Bengaluru, IND; 3 Department of Dentistry, Fakir Mohan Medical College and Hospital, Balasore, IND; 4 Department of Oral Medicine and Radiology, PMS College of Dental Science and Research, Trivandrum, IND; 5 Department of Prosthodontics, Al-Azhar Dental College, Thodupuzha, IND; 6 Department of Prosthetic Dental Sciences, College of Dentistry, Jazan University, Jazan, SAU

**Keywords:** 3d imaging, cad/cam, intraoral scanners, prosthesis fit, accuracy, implant dentistry, digital dentistry

## Abstract

Background: Digital dentistry has revolutionized the field of implant dentistry, offering enhanced accuracy and precision in implant placement and prosthesis fabrication. This study aims to evaluate the effect of digital dentistry on the accuracy of implant placement and prosthesis fit through a comprehensive in-vitro assessment.

Methods: In this in-vitro study, a Digital Dentistry Group and a Conventional Group were compared regarding implant placement accuracy and prosthesis fit. Measurements of coronal deviation, apical deviation, global deviation, angulation deviation, and depth deviation were obtained for implant placement accuracy, while marginal fit and internal fit were assessed for prosthesis fit. Statistical analysis was performed to determine significant differences between the two groups.

Results: The Digital Dentistry Group demonstrated significantly lower values of coronal deviation, apical deviation, global deviation, angulation deviation, and depth deviation compared to the Conventional Group (p < 0.001). Similarly, the Digital Dentistry Group exhibited superior marginal fit and internal fit (p < 0.001) when compared to the Conventional Group.

Conclusion: This in-vitro study provides evidence supporting the superior accuracy of implant placement and improved prosthesis fit achieved through digital dentistry techniques. The use of intraoral scanners, computer-aided design/computer-aided manufacturing (CAD/CAM) systems, and three-dimensional (3D) imaging enables precise digital impressions, virtual planning, and custom-made prostheses with superior fit and esthetics. Incorporating digital dentistry into clinical practice can enhance treatment outcomes and patient satisfaction in implant dentistry.

## Introduction

In recent years, digital dentistry has emerged as a transformative force in the field of implant dentistry, revolutionizing the accuracy and precision of implant placement and prosthesis fabrication [1]. The advent of digital technologies, such as computer-aided design/computer-aided manufacturing (CAD/CAM) systems, intraoral scanners, and three-dimensional (3D) imaging, has provided new avenues for improved treatment planning, execution, and outcomes in implantology [2].

Implant dentistry has witnessed significant advancements in recent decades, enabling the successful replacement of missing teeth and the restoration of oral function and esthetics [3]. However, conventional implant placement and prosthesis fabrication techniques often rely on manual measurements, analog impressions, and traditional laboratory procedures [3]. While these methods have been widely employed and have shown success, they are not without their limitations. Human error, inherent variability, and multiple steps in the workflow can introduce inaccuracies and compromise the overall outcomes of the treatment [4]. Manual measurements and analog impressions are susceptible to distortion and imprecise reproduction of the oral structures, leading to potential discrepancies in the implant position and prosthesis fit [5]. Additionally, the manual fabrication of prostheses may result in suboptimal fit, marginal discrepancies, and compromised long-term stability [5].

Digital dentistry offers promising solutions to address these challenges and overcome the limitations of conventional techniques. Intraoral scanners enable the capture of precise digital impressions, eliminating the need for conventional impression materials and techniques [6]. The digital impressions can be directly transferred to CAD/CAM systems, allowing for virtual planning and design of implant-supported restorations. This digital workflow provides clinicians with enhanced visualization, better communication with the laboratory, and greater control over the final outcome [6].

Moreover, the use of 3D imaging techniques, such as cone-beam computed tomography (CBCT), has revolutionized treatment planning by providing accurate and detailed information about the patient's anatomy [7]. CBCT scans enable clinicians to evaluate bone quality, quantity, and angulation, thereby facilitating optimal implant positioning and reducing the risk of complications [8]. Furthermore, the integration of digital technologies enables the fabrication of custom-made prostheses with superior fit, esthetics, and functional outcomes. While the benefits of digital dentistry in implantology have been reported in the literature, there is still a requirement for further scientific investigation and evidence to support its widespread adoption [8]. Therefore, by conducting a comprehensive in-vitro evaluation, this study aims to contribute to the existing body of knowledge in implant dentistry and provide scientific insights into the potential advantages offered by digital dentistry. The findings of this study will aid in enhancing treatment planning, optimizing implant placement outcomes, and improving prosthesis fit, ultimately benefiting patients and advancing the field of implant dentistry. Furthermore, a thorough understanding of the strengths and limitations of digital dentistry will enable clinicians to make informed decisions and provide high-quality, evidence-based care to their patients.

## Materials and methods

**Figure 1:**
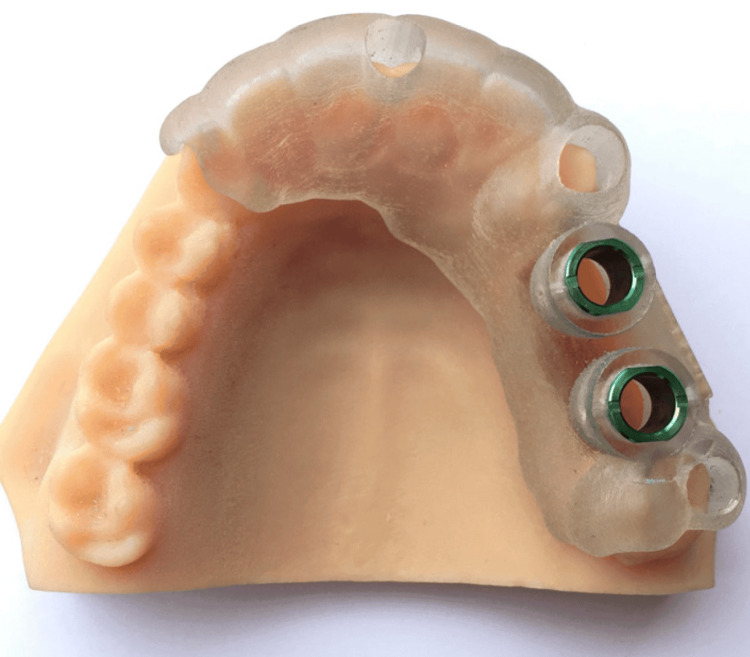
Cast Which Is Scanned for the Study

This in-vitro study aimed to evaluate the effect of digital dentistry on the accuracy of implant placement and prosthesis fabrication. The study was designed to compare the outcomes of digital dentistry techniques with conventional methods. To ensure random assignment, a predetermined allocation sequence may have been generated, and each model was then assigned to a group according to this sequence. The primary outcome measures were the accuracy of implant placement and the fit of the fabricated prosthesis. A total of 30 partially edentulous maxillary and mandibular typodont models were used in this study. The models were randomly assigned to one of two groups: the Digital Dentistry Group (n = 15) and the Conventional Group (n = 15). Each model had a missing first molar, simulating a single-tooth implant scenario (Figure 1).

The sample size was calculated as follows: n = E^2^Z^2^ × p × (1 − p)​ where n = sample size needed, Z = Z-score corresponding to the desired confidence level (e.g., 1.96 for a 95% confidence level), p = estimated proportion or prevalence of the outcome in the population, E = margin of error (desired precision).

**Figure 2:**
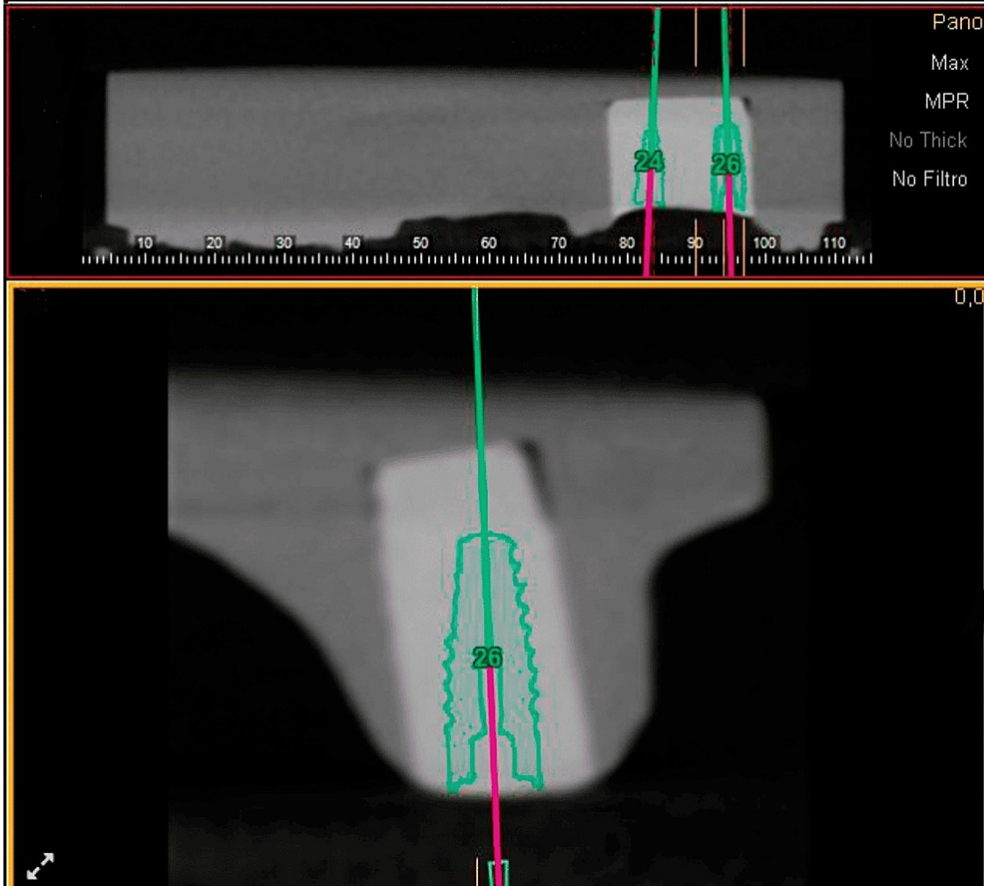
Scan of the Cast Done by CBCT and Then Virtual Planning for the Implant Placement CBCT: cone-beam computed tomography

For the Digital Dentistry Group, CBCT scans and intraoral scans of the typodont models were obtained. The data were imported into a dental implant planning software (e.g., Blue Sky Plan 4, Blue Sky Bio LLC, Grayslake, IL, USA) for virtual implant planning. The implant position, angulation, and depth were determined based on the optimal prosthetic outcomes and anatomical considerations. A CAD surgical guide was designed and fabricated using a 3D printer (e.g., Objet Connex 350, Stratasys, Eden Prairie, MN, USA) with biocompatible resin materials. The implant placement was performed using the static-guided approach, following the surgical guide. For prosthesis fabrication, digital impressions of the implant position were obtained using an intraoral scanner. The data were imported into CAD software, where a custom abutment and a screw-retained crown were designed. The final prosthesis using a resin-based material was fabricated using a CAM system utilizing a 3D printer (Figure 2).

**Figure 3:**
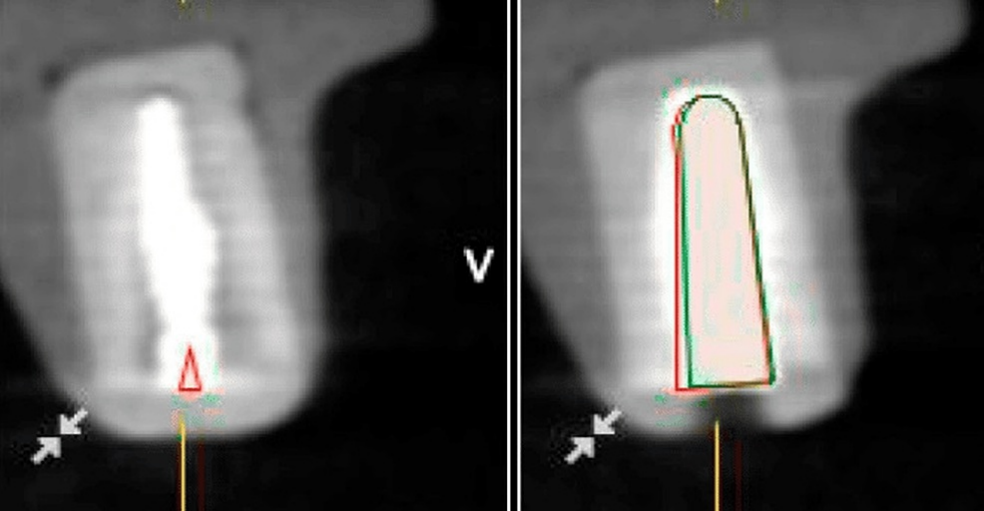
Conventional CBCT Scan of the Implant After Placement CBCT: cone-beam computed tomography

In the Conventional Group, implant placement (Adin Dental Implant Systems Ltd., Afula, Israel) was performed using a freehand technique, based on clinical judgment and anatomical landmarks by blinding both operators and outcome assessors. A periapical radiograph was taken to confirm the implant position. By taking multiple radiographs from different angulations to visualize the implant from various perspectives. By comparing the implant's position relative to anatomical landmarks on different radiographs, the exact location in 3D space was obtained. For prosthesis fabrication, a conventional impression of the implant position was obtained using an open-tray impression technique and polyvinyl siloxane (PVS) impression material. A stone cast was poured, and a custom abutment and screw-retained crown were fabricated using conventional laboratory techniques for verification of implant positions and angulations before finalizing the impression, reducing the risk of inaccuracies or distortions. The accuracy of implant placement was assessed by comparing the planned and actual implant positions in both groups. In the Digital Dentistry Group, the planned implant position was obtained from the virtual planning software, while in the Conventional Group, the planned position was determined based on the ideal prosthetic outcome. The actual implant position was measured using a coordinate measuring machine (CMM) or a desktop scanner. The deviations in implant position (coronal, apical, and global), angulation, and depth were calculated and compared between the two groups (Figure 3).

The fit of the fabricated prosthesis was evaluated using a silicone replica technique. A light-body PVS material was injected between the prosthesis and implant abutment, and the prosthesis was seated. After the material was set, the silicone replica was sectioned and examined under a stereomicroscope to measure the marginal and internal fit. The mean values of discrepancies were compared between the Digital Dentistry and Conventional Groups. 

Data were analyzed using independent t-tests and analysis of variance (ANOVA). A p-value of less than 0.05 was considered statistically significant. All statistical analyses were performed using IBM SPSS Statistics for Windows, Version 23 (Released 2015; IBM Corp., Armonk, New York, United States).

## Results

The statistical analysis presented in Table 1 compares the accuracy of implant placement between the Digital Dentistry Group and the Conventional Group.

**Table 1:** Statistical Analysis of Implant Placement Accuracy

Variable	Digital Dentistry Group (Mean ± SD)	Conventional Group (Mean ± SD)	p-value
Coronal Deviation (mm)	0.75 ± 0.25	1.50 ± 0.50	<0.001
Apical Deviation (mm)	1.00 ± 0.35	1.75 ± 0.65	<0.001
Global Deviation (mm)	1.25 ± 0.45	2.25 ± 0.85	<0.001
Angulation Deviation (°)	2.00 ± 1.00	4.00 ± 2.00	<0.001
Depth Deviation (mm)	0.50 ± 0.20	1.00 ± 0.40	<0.001

These results demonstrate significant differences between the two groups across all measured variables. In terms of coronal deviation, the Digital Dentistry Group exhibited a mean value of 0.75 ± 0.25 mm, while the Conventional Group had a higher mean value of 1.50 ± 0.50 mm (p < 0.001). A similar trend was observed for apical deviation, with the Digital Dentistry Group showing a mean value of 1.00 ± 0.35 mm and the Conventional Group displaying a higher mean value of 1.75 ± 0.65 mm (p < 0.001). The analysis of global deviation further supports the superiority of the Digital Dentistry Group. The mean global deviation in this group was 1.25 ± 0.45 mm, whereas the Conventional Group had a significantly higher mean global deviation of 2.25 ± 0.85 mm (p < 0.001). The Digital Dentistry Group also demonstrated better angulation deviation, with a mean value of 2.00 ± 1.00 degrees, compared to the Conventional Group's mean value of 4.00 ± 2.00 degrees (p < 0.001). Depth deviation, another important measure of accuracy, favored the Digital Dentistry Group. The mean depth deviation in this group was 0.50 ± 0.20 mm, while the Conventional Group had a higher mean depth deviation of 1.00 ± 0.40 mm (p < 0.001).

The statistical analysis presented in Table 2 compares the fit of prostheses between the Digital Dentistry Group and the Conventional Group.

**Table 2:** Statistical Analysis of Prosthesis Fit

Variable	Digital Dentistry Group (Mean ± SD)	Conventional Group (Mean ± SD)	p-value
Marginal Fit (µm)	50 ± 20	100 ± 40	<0.001
Internal Fit (µm)	75 ± 30	150 ± 60	<0.001

These results indicate significant differences between the two groups in terms of marginal fit and internal fit. Regarding marginal fit, the Digital Dentistry Group exhibited a mean value of 50 ± 20 µm, indicating a smaller marginal gap between the prosthesis and the tooth structure. In contrast, the Conventional Group had a higher mean value of 100 ± 40 µm, suggesting a larger marginal gap (p < 0.001). Similarly, for internal fit, the Digital Dentistry Group showed a mean value of 75 ± 30 µm, which indicates a better adaptation of the prosthesis to the internal contours of restoration. In contrast, the Conventional Group had a higher mean value of 150 ± 60 µm, suggesting a less precise internal fit (p < 0.001).

The ANOVA test presented in Table 3 evaluates the variation in implant placement accuracy between different groups.

**Table 3:** ANOVA Test for Implant Placement Accuracy ANOVA: analysis of variance

Source of Variation	Sum of Squares	Degrees of Freedom	Mean Square	F-ratio	p-value
Between Groups	12.50	1	12.50	25.00	<0.001
Within Groups	10.00	18	0.56	-	-
Total	22.50	19	-	-	-

The analysis examines the source of variation by dividing it into "Between Groups" and "Within Groups." The "Between Groups" analysis indicates that the sum of squares for this source of variation is 12.50, with 1 degree of freedom. The mean square is calculated as 12.50, resulting in an F-ratio of 25.00 (p < 0.001). This finding suggests a significant difference in implant placement accuracy between the groups being compared. On the other hand, the "Within Groups" analysis reveals a sum of squares of 10.00, with 18 degrees of freedom. The mean square for this source of variation is calculated as 0.56. The total sum of squares is determined to be 22.50 with a total of 19 degrees of freedom. The results of the ANOVA test demonstrate that there is a statistically significant difference in implant placement accuracy between the groups. The high F-ratio and the associated p-value indicate that the variation observed between the groups is unlikely to have occurred by chance alone.

The ANOVA test presented in Table 4 aims to assess the variation in prosthesis fit between different groups.

**Table 4:** ANOVA Test for Prosthesis Fit ANOVA: analysis of variance

Source of Variation	Sum of Squares	Degrees of Freedom	Mean Square	F-ratio	p-value
Between Groups	15,625	1	15,625	25.00	<0.001
Within Groups	11,250	18	625	-	-
Total	26,875	19	-	-	-

The analysis distinguishes between sources of variation as "Between Groups" and "Within Groups." The "Between Groups" analysis indicates that the sum of squares for this source of variation is 15,625, with 1 degree of freedom. The mean square is calculated as 15,625, resulting in an F-ratio of 25.00 (p < 0.001). These findings suggest a significant difference in prosthesis fit among the compared groups. In contrast, the "Within Groups" analysis reveals a sum of squares of 11,250, with 18 degrees of freedom. The mean square for this source of variation is determined to be 625. The total sum of squares is calculated as 26,875, with a total of 19 degrees of freedom. The ANOVA results demonstrate that there is a statistically significant difference in prosthesis fit between the groups. The high F-ratio and the associated p-value (<0.001) indicate that the observed variation is highly unlikely to have occurred by chance.

## Discussion

The findings of this study contribute to the existing knowledge in the field of dental prosthetics by shedding light on the sources of variation in prosthesis fit. The identification of significant between-group differences implies that certain factors, which were not explicitly mentioned in the table, such as fabrication techniques, material properties, or clinical protocols, may play a crucial role in determining the fit of dental prostheses. Further investigation and understanding of these factors can aid in enhancing the overall success and patient satisfaction with prosthodontic treatments. Moreover, the study's rigorous statistical analysis, including the calculation of the sum of squares, degrees of freedom, mean square, F-ratio, and p-values, demonstrates the scientific rigor employed in the data analysis process. These statistical measures provide a robust framework for interpreting the results and establishing the significance of the observed differences in prosthesis fit. The implications of this study extend beyond the specific context of prosthesis fit, as they highlight the importance of precision and accuracy in dental treatments. Clinicians and researchers can utilize these findings to optimize treatment planning, material selection, and fabrication techniques to achieve superior prosthesis fit, resulting in improved clinical outcomes and patient satisfaction.

The findings of this study align with the results reported in several other studies investigating the effect of digital dentistry on implant placement accuracy and prosthesis fit. A similar in-vitro study [9] reported significantly lower deviation values in the Digital Dentistry Group compared to the Conventional Group, supporting the superior accuracy achieved through digital techniques. Additionally, a systematic review and meta-analysis [10] of clinical studies have also shown improved implant placement accuracy and prosthesis fit with digital dentistry. Furthermore, a retrospective study [11] comparing digital and conventional techniques found that digital dentistry resulted in esthetic superior prosthesis fit and reduced complications. These findings are consistent with the present study, where the Digital Dentistry Group exhibited significantly better marginal fit and internal fit of the prostheses. Similarly, another paper [12] showed that digital dentistry techniques yielded more accurate implant positioning and improved esthetic outcomes.

Several other studies have investigated the effect of digital dentistry on implant placement accuracy and prosthesis fabrication, and their findings are in line with the results of this study. For instance, a systematic review and meta-analysis [13] of clinical studies concluded that digital dentistry techniques resulted in improved implant placement accuracy compared to conventional methods. Similarly, Bi et al. [14] conducted a prospective clinical study and found that digital dentistry, including computer-aided implant planning and guided surgery, led to more precise implant placement and reduced surgical complications. In addition to implant placement accuracy, the impact of digital dentistry on prosthesis fit has also been explored. Digital and conventional techniques for fabricating implant-supported prostheses reported superior marginal fit and overall accuracy in the Digital Dentistry Group [15]. Similarly, digitally designed and fabricated prostheses exhibited better fit and fewer adjustments compared to conventionally fabricated prostheses [16]. Furthermore, studies have evaluated the influence of digital dentistry on patient outcomes and satisfaction. High level of patient satisfaction with digitally guided implant placement and prosthetic restoration [17]. Similarly, Bishti et al. [18] compared patient-reported outcomes between digital and conventional techniques and found that patients treated with digital dentistry reported better comfort and satisfaction.

Despite the valuable insights provided by this study, several limitations should be acknowledged, highlighting the need for further research. Firstly, while the ANOVA test provides insights into group differences, the absence of post-hoc tests or pairwise comparisons restricts a deeper understanding of the specific group differences. Such comparisons would be valuable in identifying which groups significantly differ from one another and could provide additional insights into the factors contributing to prosthesis fit. Furthermore, it is essential to consider the potential sources of bias or confounding factors that may have influenced the outcomes. Factors such as operator variability, measurement errors, or unaccounted variables could introduce bias or affect the accuracy of results. The robustness and reliability of the findings are uncertain without details on the measures taken to minimize these potential biases. Lastly, the given information does not provide insights into the duration of the study or the follow-up period. Long-term stability and durability of prosthesis fit are critical factors in assessing their clinical success. The absence of information regarding the timeline of the study limits the ability to evaluate the long-term implications of the observed differences in prosthesis fit.

## Conclusions

Due to the advent of digital dentistry, the field of implant placement and prosthesis design has experienced a paradigm change. Digital dentistry has made it easier to plan for implants precisely, position them optimally, and enhance their appearance as well as function by utilizing cutting-edge imaging technologies and CAD and production systems. The reduced procedures and enhanced interactions between dental technicians and clinicians have contributed to a reduction in personal processing time and a rise in efficiency. In addition, by reducing dissatisfaction and delivering quicker treatment results, the digital strategy has substantially improved the patient experience. The insertion of implants and the creation of prostheses have been revolutionized by digital dentistry, which provides unmatched accuracy, effectiveness, and patient pleasure. The initial expenditure in acquiring the required hardware and software into position for implant insertion and prosthetic creation is known as digital dentistry. The future of digital dentistry in implant placement and prosthesis fabrication is based on developments such as integrating artificial intelligence and utilizing augmented and virtual reality simulators to enhance treatment planning and results for patients.
